# The influence of patients’ beliefs about medicines and the relationship with suboptimal medicine use in community-dwelling older adults: a systematic review of quantitative studies

**DOI:** 10.1007/s11096-024-01727-9

**Published:** 2024-05-05

**Authors:** Eman Rafhi, Malath Al-Juhaishi, Ieva Stupans, Julie E. Stevens, Joon Soo Park, Kate N. Wang

**Affiliations:** 1 Pharmacy, School of Health and Biomedical Sciences, RMIT University, Bundoora, VIC 3083 Australia; 2 School of Allied Health, The University of Western Australia, Crawley, WA 6009 Australia; 3 Pharmacy Department, Alfred Health, Melbourne, VIC 3000 Australia; 4 School of Engineering, Information Technology and Physical Sciences, Federation University Australia, Ballarat, VIC 3350 Australia; 5 Adelaide Medical School, Faculty of Health & Medical Sciences, University of Adelaide, Adelaide, SA 5005 Australia; 6 Clinical and Health Sciences, University of South Australia, Adelaide, SA 5000 Australia

**Keywords:** Aged, Attitude, Medication adherence, Older adults, Patient beliefs, Potentially inappropriate medication, Polypharmacy

## Abstract

**Background:**

Medication use in older adults is increasing, therefore, reducing the risk of suboptimal medicine use is imperative in achieving optimal therapeutic outcomes. Research suggests that factors such as personal beliefs and beliefs about medicines may be associated with non-adherence and inappropriate medicine use.

**Aim:**

To systematically review and identify quantitative research on the influence of beliefs about medicines and the relationship with suboptimal medicine use in older adults.

**Method:**

Searches were conducted on PubMed, EMBASE, CINAHL, and PsycINFO for quantitative studies (inception to March 2023). Inclusion criteria: (1) exposure: participants’ beliefs (personal, cultural, and medication-related), (2) outcomes: polypharmacy, potentially inappropriate medicines use, or non-adherence, and (3) participants: community-dwelling adults 65 years or above. Study selection, data extraction and quality appraisal (Joanna Briggs Institute critical appraisal checklist) were completed independently by two investigators. Data were combined in a narrative synthesis and presented in a summary of findings table.

**Results:**

Nineteen articles were included: 15 cross-sectional and four cohort studies. Outcomes of included papers were as follows; adherence (*n* = 18) and potentially inappropriate medicine use (*n* = 1). Ten studies found stronger beliefs in the necessity of medicines and/or fewer concerns led to better adherence, with one paper contradicting these findings. Three studies did not find associations between adherence and beliefs. One study confirmed an association between unnecessary drug use and a lack of belief in a "powerful other" (e.g. doctor).

**Conclusion:**

Further investigation is necessary to (1) ascertain the importance of necessity or concern beliefs in fostering adherence and, (2) examine the influence of beliefs on polypharmacy and inappropriate medicine use.

**Supplementary Information:**

The online version contains supplementary material available at 10.1007/s11096-024-01727-9.

## Impact statements

- The relationship between medication beliefs and adherence is multifaceted and can vary based on patient populations and other contextual factors.
- Stronger beliefs in the necessity of medications and reduced concerns appear to positively influence medication adherence in older adults, however further investigation is needed to ascertain the relative importance of necessity or concern beliefs in fostering adherence.
- There is a paucity of research on the influence of beliefs on polypharmacy and inappropriate medication use. Consequently, further research is needed.

## Introduction

The number of medicines prescribed and utilised by older adults is increasing [1, 2]. Inappropriate use of medicines can result in treatment failure and can increase the risk of adverse events [3]. As such, inappropriate and suboptimal medicine use are significant safety concerns in this aging population [1, 4, 5]. Therefore, appropriate use of medicines is crucial in achieving optimal therapeutic outcomes.

Suboptimal medicine use can arise from various factors, including polypharmacy, non-adherence and the prescribing of potentially inappropriate medicines (PIMs) [5]. In a tertiary hospital setting, PIM use among older adults was found to be 55%, with 6% of all admissions attributed to PIM use [6]. Current research suggests that beliefs about medicines and personal beliefs may be associated with non-adherence and therefore suboptimal medicine use [3, 7–9].

Beliefs can be influenced by an individual’s personal and cultural beliefs, as well as those shaped by personal experiences [10]. Negative experiences can result from instances of adverse drug events, medication-related burden, polypharmacy and dissatisfaction with the healthcare system [11, 12]. Moreover, a 2016 review of qualitative studies explored patients' lived experiences with medications, revealing that medication-related burden significantly influences patients' well-being, beliefs and behaviours toward medicines [13]. The rapport and trust established between patients and their healthcare providers are also pivotal factors known to impact medication use [14]. Patients often develop their own experiences, beliefs, and perceptions of their medications, which can subsequently shape their attitudes towards healthcare and the utilisation of medicines. Research also suggests that patients with a stronger belief in the necessity of their medicines with fewer concerns, demonstrate higher adherence to medicines with beliefs being identified as the most influential predictor of adherence [12, 15].

In this review, the term "beliefs" encompasses a broad spectrum of perspectives, including personal, cultural, and medication-related beliefs. The current literature employs various methods to measure beliefs, including use of validated tools such as the Beliefs About Medicines Questionnaire (BMQ) and the Beliefs and Behaviour Questionnaire (BBQ) [16, 17], with the BMQ being the most commonly utilised tool. The BMQ is an 18-item scale divided into two parts: BMQ-Specific and BMQ-General. The BMQ-Specific includes necessity and concern subscales, while the BMQ-General includes overuse and harm subscales. Each domain is rated on a five-point Likert scale, with higher scores reflecting stronger beliefs in that domain [16]. However, for this review, beliefs were not restricted to assessment solely through validated tools. Use of other methods to evaluate participants' beliefs may also be suitable for inclusion. As for the outcomes—polypharmacy, potentially inappropriate medicines use, and nonadherence—these have been defined in Box 1. By delineating these outcomes, we aim to provide a comprehensive understanding of the relationship between beliefs and various forms of suboptimal medicine use.

While systematic reviews have explored the impact of beliefs on adherence [18–23], few delve into its influence in older adults 65 years and above, resulting in their underrepresentation. Although existing research consistently demonstrates that stronger beliefs in the necessity of medicines with fewer concerns lead to better adherence [12, 15, 21, 23], reviews vary in terms of populations, methodologies, and outcomes assessed. For instance, a 2014 review highlighted the profound influence of beliefs on adherence, however it encompassed participants of all ages and was limited to qualitative studies [22]. Similarly, a 2013 review focused solely on beliefs measured through the BMQ necessity-concern framework and did not restrict participants to those aged 65 or older [21]. Nonetheless, while a more quantitative based review has been adopted, whereby medication adherence was higher where necessity beliefs were stronger, the review similarly was not restricted to older adults and only included participants diagnosed with hypertension [23]. Lastly, a 2006 review investigated the influence of beliefs on medication adherence; however, it solely focused on adherence as the single outcome measure and included adults aged 50 or above [20]. Furthermore, existing reviews fail to explore various other forms of suboptimal medicine use and lack a quantitative approach. This review exclusively incorporates quantitative studies. By identifying these beliefs, healthcare professionals may tailor adherence strategies to align with patients' individual beliefs. This personalized approach may hold the potential to enhance medication adherence and overall health outcomes in older adults.

The extent to which beliefs may influence suboptimal medicines use in older adults warrants further research, as highlighted by the reasons above. We present the first systematic review of quantitative studies which investigated the influence of beliefs on multiple forms of suboptimal medicines use (adherence, polypharmacy and inappropriate use of medicines such as PIMs) in community-dwelling older adults 65 years and above.

## Aim

To systematically review and identify quantitative research on the influence of beliefs about medicines and the relationship with suboptimal medicine use in older adults.

## Method

### Protocol and registration

The PEO (Population, Exposure, Outcome) framework was used in the development of this systematic review and reported in accordance with the Preferred Reporting Items for Systematic Review and Meta-Analysis Protocols (PRISMA) [24, 25]. The protocol was registered on The International Prospective Register of Systematic Reviews (PROSPERO) (Registration number: CRD42023416431).

### Sources and search strategy

A full electronic literature search was conducted on PubMed, EMBASE, CINAHL, and PsycINFO from inception to March 2023. The search strategy was developed in consultation with a research librarian and incorporated combinations of keywords and indexed terms (e.g. MeSH, Emtree subheadings and the APA Thesaurus of Psychological Index Terms) related to beliefs, older adults and suboptimal medicine use. Relevant keywords included: ‘health beliefs’, ‘health views’, ‘health attitude’, ‘medicine beliefs’ and ‘polypharmacy’ utilised in conjunction with Boolean operators (e.g., OR, AND) and truncations. The full detailed search strategy is available in Supplementary material 1. Based on each database, a separate strategy was developed to account for variations in indexed terms. Reference lists of included studies were also searched, and relevant journals were manually inspected.

### Eligibility criteria

Original, peer-reviewed research studies were included with the exclusion of grey literature, literature reviews, qualitative studies and studies where full articles could not be obtained. Studies were included if (1) the exposure involved participants’ beliefs including personal beliefs, cultural beliefs, health/medication related beliefs and/or attitudes, (2) the main outcome was one of three forms of suboptimal medicine use; polypharmacy, potentially inappropriate medicine use, or non-adherence, (3) participants were considered older adults 65 years or above, (4) participants were community-dwelling, (5) the study was quantitative and (6) full-text articles were available in English. The outcomes and exposures have been defined as presented in Box 1. Where full-text articles could not be obtained, authors of the study were contacted. If full-text articles still could not be obtained, the study was excluded.

**Box 1:** Definition of outcomes and exposures

Variable	Definition	
Outcomes	The main outcome was suboptimal medicine use including polypharmacy, potentially inappropriate medicines use and non-adherence at a single point in time. The absence of appropriate medicines was not screened in this review. This review only screens for the presence of suboptimal medicine use.	*Polypharmacy:* The use of greater than or equal to 5 medicines [5].
		*Potentially inappropriate medicines:* (1) High-risk medicines (e.g. Potentially Inappropriate Medicines, PIMs) which should be avoided in older adults due to their high risk of harm in this population as identified in either the Beers Criteria and/or STOPP criteria [49, 50]. (2) The identification of inappropriate medicines where participants were using medicines for an inappropriate indication or where its use was against current guidelines.
		*Non-adherence:* The process whereby a patient is not taking their medicine as advised by their prescriber [51].
Exposure	*Participants' beliefs:* Inclusion of personal beliefs, cultural beliefs, health/medication-related beliefs and attitudes.	Presented as a measure through use of validated tools such as the Beliefs about Medicines Questionnaire (BMQ) [16], however, beliefs were not limited to assessment through only this tool. Use of other methods to evaluate participants beliefs may also have been appropriate for inclusion in this review.

### Participants and population

The population of interest was community-dwelling older adults aged 65 years and above, defined, for the purpose of this review, as adults aged 65 years or above living independently in the community. Studies were included if (1) the mean age of recruited participants was 65 years or above or (2) where certain studies included older adults, the data from participants aged 65 years or older could be extracted.

### Study selection

Following export of studies to Endnote Version 21, deduplication was completed. Titles and abstracts were independently screened by two reviewers (ER, MA) for eligibility and then full-text screened again by two reviewers (ER, MA). Following review of each full-text paper, reasons for exclusion were noted in an Excel spreadsheet for comparison. Any disagreements were resolved through discussion of each paper. Where both reviewers could not come to an agreement, a third reviewer facilitated consensus.

Using the PRISMA guidelines, a flow diagram was generated to illustrate the selection process and the final studies included.

### Data extraction and synthesis

Data from papers identified to fit the inclusion criteria were extracted using a standardized data-extraction tool. Data extracted included study design, aim, country, clinical setting, number of participants, measurement of beliefs, participant eligibility criteria, exposure, outcome/s, covariates considered in analysis and the main findings of each study. As such, a narrative synthesis approach was utilised, and a summary of findings table presented. A narrative synthesis has been defined as “an approach to the systematic review and synthesis of findings from multiple studies that relies primarily on the use of words and text to summarise and explain the findings of the synthesis” [26]. Included studies were subsequently analysed and categorised based on their outcome/s; adherence, polypharmacy and/or inappropriate medicine use.

### Quality and risk-of-bias assessment

To assess the quality and risk of bias of each study, the Joanna Briggs Institute (JBI) critical appraisal checklist tool was used. This checklist serves as a tool to guide the assessment of the quality and potential inclusion of each study. It includes various appraisal checklists tailored to different types of studies. Hence, depending on the study design being evaluated, a specific checklist from the JBI critical appraisal tool was utilized.

Two reviewers (ER, MA) independently appraised each article, after having set standards for each appraisal standard. Any disagreements among reviewers were resolved through discussion. Where both reviewers could not come to an agreement, a third reviewer would facilitate consensus.

For this systematic review, a high-quality study was defined as a score of ≥ 6 out of 8 for cross-sectional studies and ≥ 8 out of 11 for cohort studies [27].

## Results

### Study selection

**Fig. 1:**
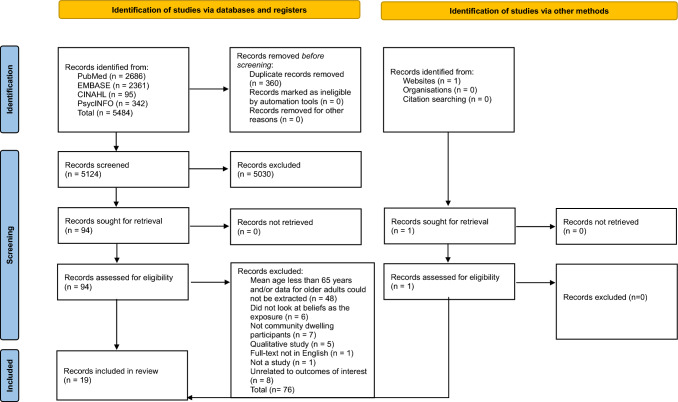
PRISMA (Preferred Reporting Items for Systematic Reviews and Meta-Analyses) flowchart of study selection process

A total of 5,484 entries were identified across four databases (Fig. 1). After eliminating duplicates (*n* = 360), 5124 articles underwent title and abstract screening. Of these, 94 underwent full-text screening, and 18 studies were included in this systematic review. A hand-search contributed one additional paper, resulting in a total of 19 studies. Of the 19 studies, 15 were cross-sectional [28–42] and four were cohort studies [43–46]. Characteristics of each included study are presented in Table 1 and 2.

**Table 1:** Characteristics of included studies

Author and year	Design & Country of study	Aim	Clinical setting & No. of participants	Eligibilty	Exposure	Outcome measure	Covariate factors considered
*Non-adherence*							
Alison Phillips et al. (2013) [43]	Cohort study United States 1-month follow up	To propose and assess the utility of factors beyond patients’ initial beliefs with regards to a treatment.	Primary care clinic of an urban research hospital In-person interviews 84 Participants	All ages w/ diagnosis of hypertension	Treatment specific Health Beliefs	Treatment adherence	NR
Byrne et al. (2005) [28]	Cross sectional study Ireland	To describe the illness perceptions and beliefs about medication of patients with established coronary heart disease.	General practices 1084 Participants	Patients 80 years or less with history of myocardial infarction, angina, revascularisation by angioplasty or coronary artery bypass grafting	Illness perceptions and medication beliefs	Secondary prevention behaviour (i.e. smoking, diet, medication adherence)	NR
Capiau et al. (2020) [29]	Cross-sectional observational study Belgium	Assess implementation adherence to NOACs and to explore experiences with and beliefs about NOACs.	Community Pharmacies 766 Participants	Started taking a NOAC at least 1 year prior to inclusion, had accessible pharmacy dispensing data and spoke and read the Dutch language	Experiences and Beliefs	Adherence to NOACs	Age, sex, BMI, living situation, education, smoking, alcohol consumption, self-rated health, history of thromboembolic events and major bleeding, falls, hospitalisations, indication of NOAC therapy, frequency, duration, number of medicines used, presence of ADRs, practical issues with NOAC, BMQ-necessity score, BMQ-concerns score, appropriate knowledge of the indication of NOAC, frequency of GP and cardiologist visits.
Cicolini et al. (2016) [30]	Cross-sectional study Italy	To evaluate the association between adherence to treatment and beliefs about medications in multi-treated elderly patients.	Primary care groups 567 Participants	Age 65–80, using ≥ 4 drugs daily for ≥ 2 months, affected by more than one chronic condition among cardiovascular, neurological, respiratory, musculoskeletal, oncologic diseases, or diabetes mellitus	Beliefs and attitudes	The difference in BMQ Concerns scale score according to adherence category	Age, gender, educational level, marital status, n. of prescribed drugs, diabetes and other diseases, BMQ concern and necessity scores and, separately, BMQ groups.
Clark et al. (2016) [46]	Cross-sectional and prospective cohort study United Kingdom 2 year follow up	To identify individual patient reasons for stopping medications for osteoporosis.	Primary care general practice 3200 Participants	Those enrolled in the COSHIBA cohort – Women w/ a DOB between 1st of January 1927 and 31st December 1942	Individual patient factors	Adherence to osteoporosis medications	NR
de Vries et al. (2014) [31]	Cross-sectional study Netherlands	To assess the role of different kinds of beliefs and treatment complexity on unintentional and intentional non-adherence, and whether this differs for glucose-, blood pressure-, and lipid-lowering drugs in patients with type 2 diabetes.	General practice clinics 345 Participants	Patients with type 2 diabetes from the GIANTT-database who had been prescribed an oral glucose-lowering drug in 2005	Beliefs	Intentional and Unintentional non-adherence in T2DM patient on glucose-, blood pressure-, and lipid-lowering drugs	NR
Durand et al. (2018) [32]	Cross-sectional study Ireland	To examine predictors of long-term adherence for patients with aTRH in primary care.	GP clinics 204 Participants	Patients meeting the criteria for apparent treatment-resistant hypertension (aTRH)	Predictors of long-term medication adherence (i.e., treatment-related beliefs)	Adherence	NR
Gadkari and McHorney (2012) [33]	Cross-sectional survey The United States	(1) Study the prevalence and predictors of unintentional non-adherence; and (2) Explore the interrelationship between intentional and unintentional non-adherence in relation to patients’ medication beliefs.	Community dwelling 24,017 Participants	Panel member of the Harris Interactive Chronic Illness Panel who are aged 40 and older, resided in the U.S., and reported one of six chronic diseases	Predictors of unintentional non-adherence including medication beliefs	Non-adherence	Age, gender, race, education, income, index disease, employment status, and self-rated health.
Lu et al. (2016) [34]	Cross-sectional study China	To investigate the variables associated with adherence with antidepressants in elderly Chinese patients.	Outpatient department of a tertiary psychiatric hospital 135 Participants	(i) 60 years or over; (ii) diagnosis of a major depressive disorder; (iii) ≥ 12 weeks of continued prescriptions for any first-line antidepressant prescribed at a stable dosage	Attitudes and beliefs	Adherence to antidepressant medication	NR
Neoh et al. (2017) [35]	Cross-sectional study Malaysia	Assess medication adherence and barriers towards medicine adherence in this elderly population.	Community dwelling 79 Participants	(i) 60 years and older; (ii) prescribed with medicines, (iii) residing around urban and rural areas of Selangor and Klang Valley	Barriers towards medication adherence Beliefs	Medication use and adherence	NR
Pagès-Puigdemont et al. (2019) [36]	Cross-sectional study Spain	To compare and contrast the health associated beliefs, experiences and types of behaviour in a group of chronic patients in an urban area of Barcelona according to their level of medication adherence.	Recruited by healthcare professionals from an outpatient pharmacy service in a tertiary hospital, sixteen community pharmacies and primary care centre 612 Participants	$\ge 18$ years of age At least one chronic condition under pharmacological treatment	Health associated beliefs, experiences and types of behaviour	Adherence	NR
Qiao et al. (2020) [37]	Descriptive Cross-sectional study China	To explore the association between frailty and medication adherence by modelling medication beliefs as mediators among community-dwelling older patients.	Recruited via flyer distribution to 22 communities 780 Participants	aged ≥ 60 years, have one or more self-report chronic diseases diagnosed by a physician, have taken at least one prescription medication in the past month	Medication beliefs (necessity and concerns)	Frailty and Medication adherence	Age, sex, years of schooling, marital status, monthly income, cognitive function, multimorbidity and polypharmacy.
Rovner and Casten (2018) [39]	Cross-sectional study United States	To evaluate determinants of medication adherence and glycaemic control in blacks with diabetes and Mild Cognitive Impairment (MCI).	Community dwelling—recruited from primary care practices 143 Participants	Black, over 65 years of age with type 2 diabetes, MCI, and HbA1c ≥ 7.5%	Personal determinants	Medication adherence Glycaemic control	NR
Sirey et al. (2013) [40]	Cross-sectional study United States	To examine the relation of psychological, illness, and tangible barriers to medication adherence among older adults in a community, nonmedical setting.	Volunteers from a subset of Elderly Nutrition Program sites who are part of the Aging Services Network 299 Participants	Community-dwelling population of older adults (age $>$ 60 years) who require nutrition assistance	Psychological, illness, and tangible barriers	Medication Adherence	NR
Unni and Farris (2011) [44]	Cohort study United States 2-year follow up	To determine whether beliefs in medicines are associated with forgetfulness and carelessness in taking medications (unintentional non-adherence).	A convenience sample from an online panel who were 65 years of age or older, US residents and enrolled in Medicare Baseline: 1061 participants Follow-up: 891 participants	Members of HI, who were 65 years of age or older, US residents and enrolled in Medicare	Medication beliefs	Unintentional non-adherence	Unclear—NR
Westberg et al. (2022) [41]	Cross-sectional study Sweden	To describe primary non-adherence among stroke survivors and to assess associations between primary non-adherence to preventive drugs and beliefs about medicines.	Stroke survivors living at home 3 months after their stroke 594 Participants	All participants were people with stroke who were enrolled in The Swedish Stroke Register between December 2011 and March 2012	Preventive drugs and beliefs about medicines	Primary non-adherence	Multivariable: *Adjusted for dependence on help/support from next of kin, difficulties with memory, and each BMQ subscale.
Wu et al. (2016) [42]	Cross-sectional study China	To examine the relationship between medication adherence to bisphosphonates and self-perception of aging in elderly female patients with osteoporosis.	Patients with osteoporosis from three tertiary hospital outpatient clinics in China 245 Participants	(a) Female patients with osteoporosis at out-patient clinics, (b) prescribed regular oral BP medication (c) aged 65 years or older, and (d) resident of China, living in China for a minimum of 1 year	Self-perception of aging	Medication adherence to bisphosphonates	Sociodemographic and osteoporosis-related data.
Foley et al. (2023) [45]	Observational Cohort study Ireland 2 year follow up	To explore the multidimensional relationship between medication beliefs and adherence among people living with multimorbidity.	Community-dwelling, recruited originally from family practice settings Baseline: 812 Participants Follow-up: 515 Participants	(a) Living with multimorbidity, (b) Have at least one dispensation of a RxRisk-V medication during the 6 months preceding baseline for the associated condition and (c) Participants with two or more RxRisk-V conditions at baseline and follow-up were included in the follow-up	Medication Beliefs	Adherence	Did not control for any variables.
*Potentially inappropriate medicines and/or inappropriate medication use*							
Rossi et al. (2007) [38]	Cross-sectional study United States	To determine the prevalence and predictors of unnecessary drug use in older veteran outpatients, with a focus on patient-related factors and health beliefs.	Primary Care Clinic in Pittsburgh, Pennsylvania 128 Participants	Community-dwelling patients aged > 60 years who self-administered > 5 medications per day and had all prescribed medicines filled by the Veterans Affairs pharmacy were eligible	Patient-related factors Health beliefs	Unnecessary drug use (Defined as occasions when patients were taking > 1 drug that received an inappropriate rating for indication, effectiveness, or therapeutic duplication.)	NR

*NR* Not reported, *BMI*  body mass index, *T2DM*  Type 2 Diabetes Mellitus, *ADR* adverse drug reactions, *GP*  general practitioner, *BP* bisphosphonate, *NOACs* Non-vitamin K oral anticoagulants, *HbA1c* glycated haemoglobin blood test, *aTRH*  apparent treatment-resistant hypertension, *MCI* mild cognitive impairment, *BMQ* beliefs about medicines questionnaire, *HI  *Harris interaction (panel of residents who agree to be invited to participate in online surveys), *RxRisk-V*  Algorithm used to identify multimorbidity (45 chronic conditions), *COSHIBA*  Cohort for Skeletal Health in Bristol and Avon, *GIANTT*  Groningen Initiative to Analyse Type 2 diabetes Treatment

**Table 2:** Measurement of beliefs and findings of included studies

Author and Year	Measurement of Beliefs	Findings
Non-adherence		
Alison Phillips et al. (2013) [43]	Treatment-specific Health Beliefs Assessed using the IPQ-R and BMQ- Specific	Patients' adherence was not significantly influenced by their beliefs and experiences, even for those with weaker habits. The interaction between patients' treatment beliefs and interactions didn't yield significant results across adherence measurements, with* R*^2^ change values ranging from .001 to .02 (*p*-values of 0.83–0.14, respectively).
Byrne et al. (2005) [28]	Beliefs about Medicines BMQ and IPQ-R	Medication beliefs were significantly and independently predictive of medication adherence. Beliefs were responsible for approximately 7% of the variance in adherence scores. A stronger belief in the necessity of medication and fewer concerns about medication were predictive of higher adherence to medication.
Capiau et al. (2020) [29]	BMQ-Specific	Necessity beliefs outweighed concerns for 92.2% of patients. Between NOAC molecules, there were no significant differences found in BMQ-necessity scores or BMQ concerns scores. However, the BMQ demonstrated a positive attitude towards NOAC therapy, where necessity beliefs outweigh the concerns. There were no statistically significant differences in adherence rates between the four belief groups: Sceptical, Indifferent, Ambivalent, Accepting.
Cicolini et al. (2016) [30]	Italian version of BMQ-specific	When compared with low adherence subjects, both Necessity and Concern scales mean scores were higher among subjects with medium adherence. Subjects with higher necessity or concerns scores were more likely to report a higher level of adherence (OR: 1.61, 95% CI 1.21–2.14; and 2.02, 95% CI 1.64–2.49, respectively; both *p* < .001). Participants achieving high necessity and low concerns scores (accepting group) were less likely (OR 0.24, 95% CI 0.16–0.37; *p* < .001) to report an acceptable level of adherence than ambivalent subjects (high necessity and concerns). Patients in the *Accepting* group (high necessity; low concerns) seem less prone to adhere to therapy. Concluded that the role of patients' beliefs toward medication remains unclear, due to contrasting results.
Clark et al. (2016) [46]	BMQ-Specific	20% reported beliefs about medications as reasons for low/non-adherence such as fear of side effects or a belief that the medications would not help (low perceived necessity).
de Vries et al. (2014) [31]	Beliefs about medicines BMQ-specific	No significant differences in necessity beliefs were found between the adherers and unintentional and intentional non-adherers. Intentional non-adherers to glucose- and blood pressure-lowering drugs had more concerns about these drugs than the adherers and unintentional non-adherers, which was only statistically significant for the blood pressure-lowering drugs. Concerns seem to be associated with intentional non-adherence to especially blood pressure-lowering drugs but not with unintentional non-adherence. Beliefs about necessity showed no clear association with either type of non-adherence. In adherent and unintentional non-adherent patients, beliefs in the necessity of their medicines outweighed their concerns about medicines. This finding applied for the three therapeutic groups. For the intentional non-adherers to blood pressure- and lipid-lowering drugs, however, concerns outweighed necessity. Concluded that addressing concerns about drugs appears to be more important than stressing the necessity of treatment in patients with diabetes.
Durand et al. (2018) [32]	IPQ–R and BMQ-specific	Treatment-related beliefs and habit strength did not interact, suggesting that medication-taking habits are more predictive of adherence than treatment-related beliefs even when the habit is weak. Correlation between beliefs and adherence was insignificant (p = 0.22).
Gadkari and Horney (2012) [33]	20-item beliefs scale (created by authors)	Across the three medication beliefs (perceived need, concerns, and affordability), perceived medication need and perceived medication affordability were stronger predictors of unintentional non-adherence than perceived medication concerns. The direct effect of the three medication beliefs on unintentional non-adherence was significant. The direct effect of medication beliefs on intentional non-adherence was significant. The effect of medication beliefs on intentional non-adherence is mediated through unintentional non-adherence. Concluded that unintentional non-adherence does not appear to be random and is predicted by medication beliefs, chronic disease, and sociodemographic factors.
Lu et al. (2016) [34]	Chinese version of the BMQ-specific	A higher necessity dimension score of BMQ and lower concern dimension score of BMQ about antidepressants were significant predictors of higher adherence (*P* < 0.001 and *P* = 0.007, respectively). Concluded that specific beliefs about antidepressants can predict adherence among Chinese elderly with depressive disorder.
Neoh et al. (2017) [35]	BMQ-Specific	Medication adherence was negatively correlated (r = –0.5) with the concerns score (P < 0.001). Majority of participants held positive beliefs about the necessity of their medications and 50.6% (n = 40) reported high medication adherence. Concluded that better adherence was significantly associated with lesser concern for the potential adverse effect of medication use.
Pagès-Puigdemont et al. (2019) [36]	Unspecified 17-item health belief statements	Bivariate analysis showed differences in 23 out of the 37 statements about patients’ health beliefs, health experiences and health behaviours between adherent and non-adherent groups. Results indicated that beliefs, experiences and behaviours have a strong impact upon medication adherence. Multivariate analysis found older age (OR 1.02, 95% CI 1.00–1.03, P = 0.022) and the statements ‘My doctor periodically reviews my treatment’ (OR 1.31, 95% CI 1.04–1.65, P = 0.021) and ‘I am motivated to continue with the treatment’ (OR 1.26, 95% CI 1.03–1.55, P = 0.028) to be significant in relation to medication adherence.
Qiao et al. (2020) [37]	BMQ-specific	Medication adherence was positively related to medication necessity and negatively related to medication concerns. The specific indirect effect through medication concerns was significantly larger than that through medication necessity. The detrimental effect of medication concerns as a mediator of frailty on medication adherence surpassed the positive effect of medication necessity, which resulted in poor adherence among frail older patients. Higher medication concerns were related to poorer medication adherence, while higher medication necessity was associated with better medication adherence.
Rovner and Casten (2018) [39]	BMQ	Compared to adherent participants, nonadherent participants had scored higher on the BMQ-Specific Concerns subscale, the BMQ-General Harm subscale, and the Diabetes Distress emotional burden subscale. Negative beliefs about medications, the emotional burden of living with diabetes, worse daily functioning, and ability to afford medications were related to suboptimal medication adherence.
Sirey et al. (2013) [40]	BMQ-Specific	Nonadherent group reported greater concerns, but no difference in perceived necessity in the BMQ. In both groups, in most participants, the perceived necessity of medications was greater than the concerns. However, adherent individuals reported higher risk/benefit scores than those individuals who reported being nonadherent. The primary finding of this study was that self-reported medication nonadherence was associated with illness (having minor or major depression, more medical conditions), psychological (greater concerns than perceived benefits of medication), and tangible (difficulty opening medication bottles) barriers in unadjusted bivariate analyses. **Bivariate associations between beliefs and non-adherence**: BMQ Concerns subscale → 2.47 (0.7) | BMQ Necessity subscale → 3.66 (0.7) Found a strong relation between greater concerns about medications (e.g., adverse effects) and medication nonadherence.
Unni, and Farris (2011) [44]	BMQ-Specific	The results indicate a significant association between belief in medicines and forgetfulness and carelessness in taking medications. The study shows that while both necessity and concern beliefs in medications were significant for intentional non-adherence, only concern beliefs were significant in unintentional nonadherence. Participants reporting forgetfulness and carelessness had high levels of concern beliefs about their medications, which may be causing non-adherence. The study concluded that in older adults who were Medicare enrolees, concern belief in medicines was significant in unintentional non-adherence.
Westberg et al. (2022) [41]	Brief IPQ BMQ	The mean scores of the primary non-adherent and adherent individuals were similar across all BMQ subscales. No associations were found between primary non-adherence and beliefs about medicines. Multivariable logistic regression models displayed no associations between the BMQ-subscales and primary non-adherence.
Wu et al. (2016) [42]	*Aging Perceptions Questionnaire (APQ)* *(translated into Chinese)*	Feelings of lacking control, expectations for negative events, beliefs of illness’s chronic duration nature and its association with aging were associated with poor adherence. The study found that high medication adherence was significantly associated with low timeline (chronic)(beliefs), low control negative, high consequences positive, and low percentage of experienced changes attributed to aging. Higher scores of the chronic timeline, emotional representations, negative control, negative consequences, and aging identity were associated with lower medication adherence—as per bivariate analysis. *Note: Timeline* subscale means the beliefs about the duration and course of the illness.
Foley et al. (2023) [45]	BMQ-Specific	Adherence was higher when necessity beliefs were high and concern beliefs were low. Adherence was also higher when necessity beliefs and concern beliefs were simultaneously high, compared to when both were simultaneously low. Findings from the confirmatory analyses indicated that ensuring necessity beliefs outweigh concern beliefs, may not be sufficient for strengthening adherence. Among people with multimorbidity, an individual with high necessity and high concern beliefs (ambivalent) would have higher adherence than an individual with low necessity and low concern beliefs (indifferent). Findings suggest that the combined effects of necessity and concern beliefs are more relevant to supporting adherence in this cohort.
*Potentially inappropriate medicines and/or inappropriate medication use*		
Rossi et al. (2007) [38]	Health Locus of Control Scale Decisional balance and self-efficacy scales from the Transtheoretical Model	58.6% of the sample had > 1 unnecessary prescribed drug. Most common reasons for a medication being considered inappropriate was lack of effectiveness (41.4%), lack of indication (39.8%) and therapeutic duplication (8.6%). Factors with tendency for association (*P* < 0.20) with any unnecessary drug use included race (white), income, number of prescription medications, and lack of belief in a "powerful other" health locus of control. Individuals with more unnecessary drug use were less likely to believe that their health was determined by a "powerful other" (such as a doctor or other health care providers) than those with no unnecessary drug use. Suggesting that patients who are less "trusting" of the health care system are more likely to have unnecessary medication use. Patients' strong health beliefs did not drive the prescribing of unnecessary drugs. However, patients who were less trusting of their health to their prescribers had more unnecessary drug use. Concluded → Certain patient characteristics and health beliefs may be important factors associated with unnecessary drug use.

*NR* Not reported, *OR * odds ratio, *CI * confidence interval, *NOACs* Non-vitamin K oral anticoagulants, *IPQ-R * the revised illness perception questionnaire, *BMQ*  beliefs about medicines questionnaire, *Brief IPQ*  brief illness perception questionnaire, *APQ*  aging perceptions questionnaire

### Study characteristics

Studies were conducted in The United States (US) (*n* = 6) [33, 38–40, 43, 44], Ireland (*n* = 3) [28, 32, 45], China (*n* = 3) [34, 37, 42], Belgium (*n* = 1) [29], Italy (*n* = 1)[30], United Kingdom (UK) *(n* = 1) [46], Netherlands (*n* = 1) [31], Malaysia (*n* = 1) [35], Spain (*n* = 1) [36] and Sweden (*n* = 1) [41]. Participants were community-dwelling, recruited from a variety of clinical settings including primary care settings, community pharmacies and outpatient clinics.

Participants' beliefs were predominantly assessed using the BMQ (*n* = 15, 78.9%), either in isolation or in combination with other assessment tools [28–32, 34, 35, 37, 39–41, 43–46]. Of the 19 studies, nine employed the BMQ-Specific in English [29, 31, 32, 37, 40, 43–46], three utilised both the General and Specific scales of BMQ [28, 39, 41], three employed the Illness Perception Questionnaire–Revised (IPQ-R) [28, 32, 43], two utilised self-made belief questionnaires [33, 36], one used the Brief IPQ [41], one employed the BMQ-Specific in Chinese [34], another in Italian [30], one used the Health Locus of Control Scale with Decisional balance and self-efficacy scales from the Transtheoretical Model [38], and finally, one employed the Aging Perceptions Questionnaire in Chinese [42].

Most studies in the review focused on participants with recognized chronic conditions, but not all had strict criteria. Out of 19 studies, three did not require a formally diagnosed chronic condition [35, 38, 44]. Specific conditions studied included hypertension (*n* = 2) [32, 43], osteoporosis (*n* = 2) [42, 46], coronary heart disease (*n* = 1) [28], type 2 diabetes (*n* = 1) [31], major depressive disorder (*n* = 1) [34], and stroke (*n* = 1) [41].

Among the 18 studies where adherence was the outcome measure, majority of papers (*n* = 15) recruited participants who were receiving treatment for a chronic health condition [28–34, 36, 37, 39, 41–43, 45, 46].

### Quality appraisal

While all studies met the inclusion criteria, their quality varied. Six cross-sectional studies fulfilled all JBI criteria, scoring 8/8 [28, 30, 33, 37, 41, 42], one scored 87.5% (7/8) [29], four scored 75.0% (6/8) [31, 34, 36, 38], and the remaining four were of moderate quality, meeting 62.5% (5/8) of the criteria due to their failure in addressing potential confounding factors, as well as other criteria [32, 35, 39, 40].

Regarding cohort studies, none met all eleven of the JBI Cohort appraisal criteria. Three studies satisfied 45.5% (5/11) of the criteria [43–45]. This was due to their failure in identifying confounding factors, lacked strategies to handle such factors, didn't address incomplete follow-up, and certain criteria was deemed "not applicable" to their study. The remaining study fulfilled 27.3% of the criteria (3/11) [46]. Detailed information about the JBI appraisal is outlined in Table 3 and 4. Due to the paucity of papers, no papers were excluded based on their quality.

**Table 3:** Summary of quality assessment of cross-sectional studies

Study	Were the criteria for inclusion in the sample clearly defined?	Were the study subjects and the setting described in detail?	Was the exposure measured in a valid and reliable way	Were objective, standard criteria used for measurement of the condition?	Were confounding factors identified	Were strategies to deal with confounding factors stated?	Were the outcomes measured in a valid and reliable way?	Was appropriate statistical analysis used?	Total ‘yes’ response out of 8
Byrne et al. (2005). [28]	Yes	Yes	Yes	Yes	Yes	Yes	Yes	Yes	8
Capiau et al. (2020). [29]	Yes	Yes	Yes	Yes	Yes	No	Yes	Yes	7
Cicolini et al. (2016). [30]	Yes	Yes	Yes	Yes	Yes	Yes	Yes	Yes	8
de Vries et al. (2014). [31]	Yes	Yes	Yes	Yes	No	No	Yes	Yes	6
Durand et al. (2018). [32]	Unclear	Yes	Yes	Yes	No	No	Yes	Yes	5
Gadkari and McHorney (2012). [33]	Yes	Yes	Yes	Yes	Yes	Yes	Yes	Yes	8
Lu et al. (2016) [34]	Yes	Yes	Yes	Yes	No	No	Yes	Yes	6
Neoh et al. (2017). [35]	Yes	Yes	Yes	n/a	No	No	Yes	Yes	5
Pagès-Puigdemont et al. (2019). [36]	Yes	Yes	No	Yes	Yes	No	Yes	Yes	6
Qiao et al. (2020). [37]	Yes	Yes	Yes	Yes	Yes	Yes	Yes	Yes	8
Rossi et al. (2007). [38]	Yes	Yes	Yes	Yes	No	No	Yes	Yes	6
Rovner and Casten (2018). [39]	Unclear	Yes	Yes	Yes	No	No	Yes	Yes	5
Sirey et al. (2013). [40]	Yes	Yes	Yes	Unclear	No	No	Yes	Yes	5
Westberg et al. (2022). [41]	Yes	Yes	Yes	Yes	Yes	Yes	Yes	Yes	8
Wu et al. (2016). [42]	Yes	Yes	Yes	Yes	Yes	Yes	Yes	Yes	8

**Table 4:** Summary of quality assessment of cohort studies

Study	Were the two groups similar and recruited from the same population?	Were the exposures measured similarly to assign people to both exposed and unexposed groups?	Was the exposure measured in a valid and reliable way?	Were confounding factors identified	Were strategies to deal with confounding factors stated?	Were the groups/participants free of the outcome at the start of the study (or at the moment of exposure)?	Were the outcomes measured in a valid and reliable way?	Was the follow up time reported and sufficient to be long enough for outcomes to occur?	Was follow up complete, and if not, were the reasons to loss to follow up described and explored?	Were strategies to address incomplete follow up utilized?	Was appropriate statistical analysis used?	Total ‘yes’ response out of 11
Alison Phillips et al. (2013) [43]	Unclear	n/a	Yes	Unclear	Unclear	n/a	Yes	Yes	Yes	Unclear	Yes	5
Clark et al. (2016) [46]	No	n/a	Yes	Unclear	Unclear	No	Unclear	Yes	Unclear	No	Yes	3
Foley et al. (2023) [45]	Unclear	n/a	Yes	No	No	n/a	Yes	Yes	Yes	Unclear	Yes	5
Unni and Farris (2011) [44]	Yes	n/a	Yes	No	No	n/a	Yes	Yes	Unclear	No	Yes	5

### Outcomes of interest

While the inclusion criteria covered various aspects of suboptimal medicines use (adherence, polypharmacy, PIMs/inappropriate medicine use), most studies in this review focused on adherence as the primary outcome. Of the 19 studies, 18 investigated adherence [28–37, 39–46], one examined unnecessary medicine use (where patients were using more than one medication receiving an inappropriate rating in terms of indication, effectiveness, or therapeutic duplication) [38], and no studies explored polypharmacy as its primary outcome.

### Beliefs and inappropriate medicine use

In a US study, 58.6% of patients were prescribed more than one unnecessary medication, with reasons including ineffectiveness (41.4%), lack of medical indication (39.8%), and therapeutic duplication (8.6%) [38]. Factors including race (white), income, number of prescribed medications, and a lack of belief in a "powerful other" health locus of control were associated with unnecessary medicine use [38]. Interestingly, those with higher unnecessary medicine use were less likely to believe in external factors like physicians influencing their health [38]. While strong health beliefs did not directly lead to unnecessary drug prescribing, the study suggested that patients who have less trust in the healthcare system are more likely to engage in unnecessary medicine use [38]. Overall, patient characteristics and health beliefs were identified as potential contributors to unnecessary medicine use [38].

### Beliefs and adherence

Of the 19 studies included in this review, 18 investigated adherence [28–37, 39–46]. Of these, three studies did not discover any significant associations between patient beliefs and adherence [32, 41, 43]. However, irrespective of the tool used, 15 studies revealed that patient beliefs influenced medication adherence [28–31, 33–37, 39, 40, 42, 44–46]. Among these, 10 studies specified the importance of stronger necessity beliefs in medicine and/or fewer concerns leading to better adherence [28, 29, 33–35, 37, 39, 40, 44, 45]. One study highlighted the significant impact of health beliefs on medication adherence, noting distinct variations in these beliefs between adherent and non-adherent individuals [36]. Another revealed an association between beliefs regarding the chronic duration of an illness and decreased adherence [42]. One study presented contradictory findings, indicating that patients with higher necessity beliefs and lower concerns were less inclined to adhere to therapy compared to ambivalent subjects (high necessity and concerns) [30]. Additionally, another study highlighted that low perceived necessity of medicines contributed to non-adherence [46], while a separate study observed no significant differences in necessity beliefs between adherent and non-adherent individuals, however stronger concern beliefs were associated with intentional non-adherence [31].

Using the BMQ, nine studies found stronger beliefs in the necessity of medicine and/or fewer concerns led to better adherence [28, 29, 34, 35, 37, 39, 40, 44, 45]. Likewise, using a 20-item beliefs scale, one study found that perceived medication need and perceived medication affordability were stronger predictors of unintentional non-adherence than medication concerns [33]. While one study did not find significant differences in adherence among four belief groups, self-reported adherence was high and necessity beliefs outweighed concerns for 92.2% of patients [29]. Likewise, one study revealed that adherence was higher when necessity beliefs were high and concern beliefs were low [45]. However, adherence was also higher when both necessity and concern beliefs were high, compared to when both were simultaneously low [45]. Additionally, one study indicated that while both necessity and concern beliefs in medications were significant for intentional non-adherence, only concern beliefs were significant in unintentional non-adherence [44].

## Discussion

### Statement of key findings

To our knowledge, this is the first systematic review of quantitative studies which assesses beliefs as a contributing factor to multiple forms of suboptimal medicines use such as adherence, potentially inappropriate medicine use (including PIMs), and/or polypharmacy in older adults. Most studies indicated that patient beliefs did indeed influence medication adherence [28–30, 33–37, 39, 40, 42, 44–46]. In many instances, stronger beliefs in the necessity of medications and/or reduced concerns regarding them were associated with improved adherence. However, one paper presented conflicting results [30], another found no significant differences in necessity beliefs between adherent and non-adherent individuals [31], and others did not find any significant associations between patient beliefs and adherence [32, 41, 43]. Among the studies reviewed, no papers examined the influence of beliefs on polypharmacy, and only one investigated its influence on inappropriate medication use [38]. As such, the influence of beliefs on polypharmacy and the inappropriate use of medicines remains an underexplored area.

### Strengths and weaknesses

This systematic review undertook a comprehensive approach by encompassing various outcome measures of suboptimal medication use and did not confine measurement of beliefs to a single validated tool. Qualitative studies were excluded to maintain consistency across findings and to enhance the generalisability of results. To minimise researcher bias, study selection, data extraction and quality assessment were conducted independently by two investigators.

There were certain limitations in this review. Firstly, the review only considered studies written in English, this restricting the pool of available data. Additionally, due to limited existing literature, the review could not draw definitive conclusions regarding the influence of beliefs on polypharmacy or inappropriate medicine use.

### Interpretation of findings

Among 18 studies exploring beliefs and adherence, findings suggest that the relationship between medication beliefs and adherence is multifaceted and can vary based on patient populations and other contextual factors. Some US, Swedish, and Irish studies showed no significant association between beliefs and non-adherence [32, 41, 43]. Most studies were conducted on participants who were utilizing medications to manage long-term chronic conditions, this emphasizing the clinical significance of beliefs in managing adherence among patients diagnosed with chronic diseases. However, while most studies emphasized the importance of strong necessity and/or low concern beliefs for higher adherence, there were contradictory studies [30]. Additionally, one study aligned with previous literature, however also found higher adherence when both necessity and concern beliefs were high [45]. Beliefs are an interpersonal factor that can be influenced by individual cultural beliefs and personal experiences.

Due to the complexity of managing multiple chronic conditions and the consequent need for multiple medications, it is argued that polypharmacy in older adults can have a detrimental impact on adherence and increase morbidity [47, 48]. While this assertion holds true, it is important to note that no individual paper was identified to investigate the influence of beliefs on polypharmacy. Therefore, the association between beliefs and polypharmacy remains relatively underexplored.

While PIMs are typically defined as high-risk medicines, as outlined in the Beers or STOPP criteria [49, 50], none of the studies reviewed defined inappropriate medicines as such. One study revealed a significant prevalence (58.6%) of unnecessary medicine use, influenced by factors like the quantity of prescribed medications and patient distrust [38]. Health beliefs were also identified as a potential contributor to unnecessary medicine use [34]. However, despite the known impact of high-risk medications on older adults, the relationship between inappropriate medicine use and patient beliefs remains unclear. Among the 19 studies exploring beliefs, only one investigated their influence on inappropriate medicine use, hence further research is needed.

Current literature, supported by two studies in this review [36, 38], emphasizes the positive impact of a trusting patient–prescriber relationship on medication usage [14]. Research suggests that, (1) receiving medication reviews from doctors may correlate with better adherence [36], and (2) patients who have less trust in their healthcare providers may be more prone to unnecessary medicine use [38]. Therefore, negative experiences and distrust can negatively impact medication-taking behaviour [11]. Considering these findings, coupled with the results of this review, it is evident that patient–prescriber relationships may serve as a critical factor influencing patients' medication beliefs, subsequently affecting their adherence and medication use.

This review places emphasis on the influence of beliefs on adherence, polypharmacy, or inappropriate medicines use, however other influencing factors have been identified. Factors associated with unnecessary medicine use included race (white), income level, and number of prescribed medications [38]. Conversely, factors linked to non-adherence encompassed concerns about side effects, sociodemographic factors, chronic diseases, emotional burden, medication costs, and practical barriers (e.g. dexterity) [30, 33, 39, 40, 42, 46]. Hence, while beliefs may have an impact on suboptimal medicine use, it is crucial to recognise that other barriers may also be contributing to non-adherence.

There were limitations present within the methodology of certain studies. Of the 20 studies, only four did not require participants to have a predefined medical condition, thus narrowing the applicability of findings to specific populations. In addition, where adherence was measured, self-reported questionnaires were employed, potentially introducing bias as participants may overrepresent their adherence.

### Further research

There is a need for further research in the understanding of beliefs and its influence on polypharmacy and inappropriate medicines use. More so, additional research is warranted to more comprehensively determine whether necessity or concern beliefs play a pivotal role in adherence. Future studies should explore the benefits in reducing patient concerns and increasing necessity beliefs in improving medication adherence.

## Conclusion

Findings suggest that the relationship between medication beliefs and adherence is multifaceted and can vary based on patient populations and other contextual factors. While most studies included in this review supported the finding that stronger beliefs in the necessity of medicines and reduced concerns appear to positively influence adherence in older individuals, further investigation is needed to ascertain the relative importance of necessity or concern beliefs in fostering adherence. Additionally, there is a paucity of research on the influence of beliefs on polypharmacy and inappropriate medicine use, highlighting the need for further research in this area.

### Supplementary Information

Below is the link to the electronic supplementary material.

Supplementary file1 (DOCX 16 KB)
