# Supplementary material for: The influence of patients’ beliefs about medicines and the relationship with suboptimal medicine use in community-dwelling older adults: a systematic review of quantitative studies
Source: Int J Clin Pharm. 2024 May 5;46(4):811–30. doi: 10.1007/s11096-024-01727-9 (PMC11286706; doi:10.1007/s11096-024-01727-9)
Supplement: Supplementary file 1 — Supplementary file1 (DOCX 16 KB) [file 11096_2024_1727_MOESM1_ESM.docx]

**The influence of patients’ beliefs about medicines and the relationship with suboptimal medicine use in community-dwelling older adults: a systematic review of quantitative studies.**

Ms. Eman Rafhi ^1^ *, Mrs. Malath Jamal Al-Juhaishi ^1^, Prof. Ieva Stupans ^1^, Dr Julie Stevens ^1,5,6^, Dr. Joon Soo Park^2,4^, and Dr. Kate Wang ^1,2,3^

^1^ Pharmacy, School of Health and Biomedical Sciences, RMIT University, Bundoora, VIC 3083, Australia

^2^ School of Allied Health, The University of Western Australia, Crawley, WA 6009, Australia

^3^ Pharmacy Department, Alfred Health, Melbourne, VIC 3000, Australia

^4^  School of Engineering, Information Technology and Physical Sciences, Federation University Australia, Ballarat, VIC, 3350, Australia

^5^ Adelaide Medical School, Faculty of Health & Medical Sciences, University of Adelaide, Adelaide, SA, 5005, Australia

^6^ Clinical and Health Sciences, University of South Australia, Adelaide, SA, 5000, Australia

***** Author to whom correspondence should be addressed. E-mail: s3598938@student.rmit.edu.au

**Supplementary Material**

**Supplementary Table 1:** Search Strategy for each database

| **Search Strategy** | **Results** |
| --- | --- |
| **PubMed** | |
| (((((((((((((((((((("health beliefs") OR ("health views")) OR ("health attitude")) OR ("cultural belief")) OR (cultural view)) OR (cultural attitude)) OR (medicine beliefs)) OR (beliefs about medicine)) OR ("medication beliefs")) OR (medication view*)) OR (medicines view)) OR (medication attitudes)) OR (medicine attitudes)) OR (personal view)) OR (personal belief)) OR ("patient view")) OR ("patient attitude")) OR (patient beliefs)) OR (((culture [MeSH terms]) OR (ethnology [MeSH terms])) OR (attitude [MeSH terms]))) AND (((((Polypharmacy) OR ("potentially inappropriate medication")) OR (non adherence)) OR (non-adherence)) OR (((polypharmacy [MeSH Terms]) OR (potentially inappropriate medication list [MeSH Terms])) OR (medication adherence [MeSH Terms])))) AND ((((((((((("older adults") OR (Elderly)) OR (elderly people)) OR (65 years or above)) OR (65 years or older)) OR (Older people)) OR (Older person)) OR (Elderly person)) OR (Old person)) OR (Senior citizen)) AND (aged[MeSH Terms])) | 2,686 |
| **EMBASE** | |
| 'health beliefs'/exp OR 'health beliefs' OR 'health views' OR 'health attitude'/exp OR 'health attitude' OR 'cultural belief'/exp OR 'cultural belief' OR 'cultural view' OR 'cultural attitude' OR 'medicine beliefs' OR 'beliefs about medicine' OR 'medication beliefs' OR 'medication view*' OR 'medicines view' OR 'medication attitudes' OR 'medicine attitudes' OR 'personal view' OR 'personal belief' OR 'patient view' OR 'patient attitude'/exp OR 'patient attitude' OR 'patient beliefs' OR 'health belief'/exp OR 'patient attitude'/exp OR 'patient attitude' AND 'polypharmacy' OR 'potentially inappropriate medication' OR 'non adherence' OR 'non-adherence' OR 'polypharmacy' OR 'inappropriate polypharmacy' OR 'potentially inappropriate medication' AND 'older adults' OR 'elderly' OR 'elderly people' OR '65 years or above' OR '65 years or older' OR 'older people' OR 'older person' OR 'elderly person' OR 'old person' OR 'senior citizen' OR 'aged' | 2,361 |
| **CINAHL** | |
| "health beliefs" OR "health views" OR "health attitude" OR "cultural belief" OR cultural view OR cultural attitude OR medicine beliefs OR 'beliefs about medicine' OR "medication beliefs" OR medication OR (MH "health beliefs") OR (MH "culture") OR (MH "attitude") OR (MH "attitude to medical treatment) AND polypharmacy OR “potentially inappropriate medication” OR non adherence OR non-adherence OR (MH "polypharmacy") AND “older adults” OR elderly OR elderly people OR 65 years or above OR 65 years or older OR older people OR older person OR elderly person OR old person OR senior citizen OR (MH "aged+") OR AG 65+ | 95 |
| **PsycINFO** | |
| "health beliefs" OR "health views" OR "health attitude" OR "cultural belief" OR cultural view OR cultural attitude OR medicine beliefs OR 'beliefs about medicine' OR "medication beliefs" OR medication view* OR medicines view OR medication attitudes OR medicine attitudes OR personal view OR personal belief OR "patient view" OR "patient attitude" OR patient beliefs OR beliefs (nonreligious) OR "cultural factors" OR "cultural attitudes" OR "patient attitudes" OR "attitudes" AND polypharmacy OR “potentially inappropriate medication” OR non adherence OR non-adherence AND older adults OR elderly OR elderly people OR 65 years or above OR 65 years or older OR older people OR older person OR elderly person OR old person OR senior citizen OR older adulthood | 342 |
